# Role of N-linked glycosylation in the enzymatic properties of a thermophilic GH 10 xylanase from *Aspergillus fumigatus* expressed in *Pichia pastoris*

**DOI:** 10.1371/journal.pone.0171111

**Published:** 2017-02-10

**Authors:** Xiaoyu Chang, Bo Xu, Yingguo Bai, Huiying Luo, Rui Ma, Pengjun Shi, Bin Yao

**Affiliations:** 1 College of Biological Sciences and Engineering, Jiangxi Agricultural University, Nanchang, People’s Republic of China; 2 Key Laboratory for Feed Biotechnology of the Ministry of Agriculture, Feed Research Institute, Chinese Academy of Agricultural Sciences, Beijing, People’s Republic of China; Leibniz-Institut fur Naturstoff-Forschung und Infektionsbiologie eV Hans-Knoll-Institut, GERMANY

## Abstract

*N*-Glycosylation is a posttranslational modification commonly occurred in fungi and plays roles in a variety of enzyme functions. In this study, a xylanase (*Af*-XYNA) of glycoside hydrolase (GH) family 10 from *Aspergillus fumigatus* harboring three potential *N*-glycosylation sites (N87, N124 and N335) was heterologously produced in *Pichia pastoris*. The *N*-glycosylated *Af*-XYNA (WT) exhibited favorable temperature and pH optima (75°C and pH 5.0) and good thermostability (maintaining stable at 60°C). To reveal the role of *N*-glycosylation on *Af*-XYNA, the enzyme was deglycosylated by endo-β-N-acetylglucosaminidase H (DE) or modified by site-directed mutagenesis at N124 (N124T). The deglycosylated DE and mutant N124T showed narrower pH adaptation range, lower specific activity, and worse pH and thermal stability. Further thermodynamic analysis revealed that the enzyme with higher N-glycosylation degree was more thermostable. This study demonstrated that the effects of glycosylation at different degrees and sites were diverse, in which the glycan linked to N124 played a key role in pH and thermal stability of *Af*-XYNA.

## Introduction

Hemicellulose, is the second most abundant polysaccharide after cellulose in nature. Until now, the efficient conversion of hemicellulosic biomass in various agro-industrial processes has been received much attention [1, 2]. Xylan, the most abundant hemicellulose polymer, is composed of homopolymeric backbone substituted with side chains of different substituents. Due to the complex of xylan, which require a variety of hydrolytic enzymes to act synergistically. The branch degrading enzymes includes α-l-arabinofuranosidase, α-d-glucuronidase, acetylxylan esterase, and feruloyl or coumaroyl esterase [3, 4]. And, the backbone-hydrolyzing enzymes endo-β-1,4-xylanase and β-d-xylosidase. Among them, β-1,4-endoxylanase (EC 3.2.1.8) catalyze the hydrolysis of β-1,4-linked d-xylopyranose units from the homopolymeric backbone of xylan. According to the carbohydrate-active enzyme (CAZy) database (http://www.cazy.org/Glycoside-Hydrolases.html), xylanase are classified in the glycoside hydrolases families 5, 7, 8, 10, 11, 26, 30 and 43, and majority of xylanases are confined into GH families 10 and 11 [5].

Xylanases have widespread applications in several industries, such as animal feed, brewing, textile, pulp biobleaching, beverage, and biofuel industries [6]. So far, the most widely used xylanases from mesophilic fungi. The genera *Trichoderma*, *Aspergillus* and *Penicillium* are generally highly active over a temperature range of 40–60°C [4, 7]. Because of the lacking of excellent stability at high temperature, thus limiting their applications in animal feed and brewing industries [8]. The thermophilic and thermostable xylanases are of interest in the increasing hydrolytic rates and the reducing contamination risks in industrial processes for longer durations at high temperatures [9]. A number of thermostable xylanases, which were from thermophilic fungi including the genera *Phialophora*, *Paecilomyces*, *Humicola*, *Malbranchea*, *Myceliophthora*, and *Thielavia*, have been expressed in *Pichia pastoris* expression system for basic research and industrial production purposes [10–14].

Heterologous expression in yeast has a lot of advantages, including high expression level, production of correctly folded recombinant proteins with post-translational modification, secrete reasonable amounts of protein [15]. *Pichia pastoris* has become a useful experimental tool for large-scale expression of xylanases in heterologous protein-expression hosts and widely used in engineering and production In addition, as the most common form of posttranslational modification in eukaryotes, glycosylation can be devided into two major types, N-linked and O-linked glycosylation [16]. Recently years, N-glycosylation studies in *P*. *pastoris* have attracted increasing attention. The diverse roles of glycans have multi-effects on the conformational maturation of glycoproteins. In addition, the N-glycans on the different sites had different influences for the secretion, folding, stability, and other enzymatic properties [17].

The thermophilic fungus *Aspergillus fumigatus* is suitable for recycling lignocellulosic biomass and plays a crucial role in thermophilic enzymes production. Upon inducted with xylan, the GH 10 xylanase (GenBank accession no. XM_749010.1) was significantly up-regulated [18]. In this study, the gene *Af-xynA* coding for a GH 10 xylanase was heterologous expressed in *Pichia pastoris*. To elucidate the effect of N-glycosylation at different sites on the enzymatic properties of *Af*-XYNA, site-directed mutagenesis and enzymatic treatment were used to remove the N-glycans completely or partially. The results showed that N-glycosylated xylanase has higher activity over a broader pH range and better stability.

## Materials and methods

### Strains, media, vectors and chemicals

Strain *A*. *fumigatus* Af293 was obtained from the Fungal Genetics Stock Center, which genome has been published in 2005. *Escherichia coli* Trans1-T1 (TransGen Biotech, Beijing, China) was cultivated in Luria-Bertani (LB) medium at 37°C for gene cloning and sequencing. *P*. *pastoris* GS115 (Invitrogen, Carlsbad, CA) cultivated in yeast peptone dextrose (YPD) medium at 30°C was used for gene expression. The plasmids pGEM-T Easy (Promega, Madison, WI) and pPIC9 (Invitrogen) were used as cloning and expression vectors, respectively. Beechwood xylan, barley β-glucan, carboxymethyl cellulose sodium (CMC-Na) and locust bean gum were purchased from Sigma-Aldrich (St. Louis, MO). Soluble wheat arabinoxylan was obtained from Megazyme (Wicklow, Ireland). The DNA purification kit, LA *Taq* DNA polymerase and restriction endonucleases were purchased from TaKaRa (Otsu, Japan). SV Total RNA Isolation System was purchased from Promega.T4 DNA ligase was from New England Biolabs (Hitchin, UK). All chemicals were of analytical grade and commercially available.

### Cloning of the cDNA of *Af-xynA*

Mycelia of strain Af293 was collected after 3-day-growth in the wheat bran medium containing 0.5 g/L KCl, 5 g/L (NH_4_)_2_SO_4_, 1 g/L KH_2_PO_4_, 0.5 g/L MgSO_4_·7H_2_O, 0.1 g/L CaCl_2_, 0.001 g/L FeSO_4_·7H_2_O, and 10 g/L wheat bran at 45°C. The total RNA was extracted using the SV Total RNA Isolation System (Promega KK, Tokyo, Japan) according to the manufacturer’s instructions. cDNAs were synthesized in vitro using the TransScript^®^ One-Step Gene Removal and cDNA Synthesis SuperMix (TransGen Biotech, Beijing, China) with the total RNA as the template. The full-length cDNA of the xylanase-encoding gene (*Af-xynA*) was amplified using the specific primers (*Af-xynA* PF: 5'-GGGGAATTCGCGCCTTCGAGCAGCAAGAACGACG-3' and *Af-xynA* PR: 5'- GGGGCGGCCGCCTAGCATACAGTGCAGGGCTTGTTC-3', restriction sites underlined), which were designed based on the genomic DNA sequence (GenBank accession no. XM_749010.1). The specific PCR products were ligated into the pGEM-T Easy vector for sequencing.

### Site directed mutagenesis

The gene fragment coding for mutant N124T was constructed by using the site-directed mutagenesis. Overlap extension PCR was performed with specific primers (N124T F: 5'-GGGTCACCTCCAGGACCTGGACCGCCACAG-3' and N124T R: 5'-CACTTCTTTGAGTTCTGTGGCGGTCCAG-3'), 10 ng of native *Af-xynA*/pGEM-T plasmid, and KOD DNA polymerase (Novagen, USA). The specific PCR products were ligated into the pGEM-T Easy vector for sequencing.

### Heterologous expression in *P*. *pastoris*

The cDNA fragments of *Af-xynA* and N124T without the signal peptide-coding sequences were amplified with specific expression primers (PF and RP), digested with *Eco*RI and *Not*I, and then cloned by ligation into the pPIC9 vector in-frame fusion with the α-factor signal peptide to construct the recombinant plasmids. The resulting plasmid was transformed into *E*. *coli* DH5α, and the positive recombinant plasmid was sequenced (Beijing Sanbo Zhiyuan Biotechnology Company, Beijing, China). The identification of the correct recombinant plasmid was linearized using *Bgl*II and transformed into *P*. *pastoris* competent cells by electroporation. The positive transformants selected first in synthetic media lacking histidine were screened based on their enzymatic activities in shake tubes, and the transformant exhibiting the highest activity were selected for fermentation in a 1-L Erlenmeyer flask.

### Purification of recombinant xylanases

The cell-free culture supernatants were collected by centrifugation at 12,000 g for 10 min at 4°C, and concentrated by 5 kDa cut off Vivaflow 200 ultrafiltration membrane (Vivascience, Hannova, Germany). The crude enzymes were loaded onto the HiTrap^™^ desalting columnand HiTrap Q Sepharose XL FPLC column (GE Healthcare, Uppsala, Sweden) equilibrated with buffer A (20 mM Tris–HCl, pH 8.0). Proteins were eluted with 1.0 M NaCl in the same buffer at a flow rate of 3 mL/min. Fractions exhibiting xylanase activity were pooled, and concentrated by 5 kDa cut off Vivaflow 50 ultrafiltration membrane (Vivascience, Hannova, Germany). SDS-PAGE was performed in a 12% (w/v) polyacrylamide gel to analyze the purified enzyme. The protein concentrations were determined by a protein assay kit (Bio-Rad, USA).

### Deglycosylation of the xylanase

Progressive native deglycosylation was performed using 100 μg of the purified *Af*-XynA and N124T were added 1X GlycoBuffer 3 (50 mM sodium acetate, pH 6.0) and digested with 2μL (1000 U) endo-β-N-acetylglucosaminidase H (Endo H, New England Biolabs, USA). The reaction was carried out at 37°C for 12 h and then stopped by freezing the reaction mixtures at −80°C. Deglycosylation in denaturing conditions was performed by digesting every 100 μg of pre-denatured protein in denaturing buffer (0.5% SDS and 1.0% β-ME) at 100°C for 10 min with 1 μL (500 U) of Endo H at 37°C for 2 h using the protocol as recommended by the manufacturer. The deglycosylated enzymes were also analyzed by SDS–PAGE and compared with their glycosylated counterparts.

### Xylanase activity assay

The xylanase activity was determined with 3, 5-dinitrosalicylic acid (DNS) method [19]. The reaction system consisted of 900 μL 1% (w/v) birchwood xylan in McIlvaine buffer (pH 5.0) and 100 μL of appropriately diluted enzyme solution. The reaction mixture was incubated at 60°C for 10 min, followed by the addition of 1.5 mL of DNS reagent to terminate the reaction. The mixtures were then boiled for exactly 5 min and cooled down to room temperature. Absorbance at 540 nm was measured. One unit of xylanase activity was defined as the amount of enzyme that released 1 μmol of reducing sugar from the substrate equivalent to xylose per minute under the assay conditions. Each assay had triplicate.

### Biochemical characterization

Brichwood xylan was used as the substrate for enzyme characterization. The optimal pH for enzyme activity was determined at 60°C for 10 min in buffers over the pH range from 3.0 to 8.0. The buffers used were 100mM citric acid–Na_2_HPO_4_ (pH 3.0–8.0) and 100 mM Tris–HCl (pH 8.0). The pH stability was estimated by measuring the residual enzyme activity under the standard conditions (pH 5.0, 75°C, 10 min) after pre-incubation of the enzymes in buffers of pH 3.0–11.0 at 37°C for 1 h without the substrate.

The optimal temperature for enzyme activity was examined at pH 5.0 for 10 min over the temperature range of 50–90°C with brichwood xylan as substrate. Thermal stability of the enzyme was determined at 60°C or 70°C with pre-incubation of enzyme for 5, 10, 20, 30, and 60 min without substrate and the residual enzyme activity was measured at pH 5.0 and 75°C for 10 min.

To determine the effect of different metal ions and chemical reagents on the enzyme activity of xylanase, 5 mM of various metal ions and chemical reagents were added to the reaction system individually. The system without any additive was used as a control.

The thermodynamics (*T*_m_s) of WT and mutant N124T were analyzed by using differential scanning calorimetry (DSC) over the temperature range of 25°C to 90°C.

### Substrate specificity and kinetic parameters

Substrate specificities of the purified xylanase were investigated in the standard assay system containing one of the following substrates (1%; w/v): beechwood xylan, soluble wheat arabinoxylan, barley β-glucan, CMC-Na, and locust bean gum.

The *K*_m_ and *V*_max_ values for the purified enzymes were determined from a Lineweaver-Burk plot using the non-linear regression computer program GraFit (Version 7, Erithacus Software, Horley, UK). The enzyme activity was assayed at 60°C in McIlvaine buffer (pH 5.0) containing 0.5–2 mg/mL birchwood xylan as the substrate. Each experiment was repeated three times and each experiment included three replicates.

### Analysis of hydrolysis products

The hydrolysis products of beechwood xylan and soluble wheat arabinoxylan by purified the wild-type and the mutant enzymes were determined by using high-performance anion exchange chromatography (HPAEC; Thermo Fisher Scientific, Sunnyvale, CA) equipped with a Carbo-Pac PA200 column (3 μm × 250 mm). The reaction mixtures were incubated at pH 6.0, 37°C for 12 h, followed by 10-min boiling water bath to terminate the reaction and centrifugation at 12000 g for 5 min to remove unsolved residues. To remove the extra enzyme, the clear supernatants were centrifuged (10,000 × *g*, 4°C, 10 min) through a 3-kDa Amicon Ultra centrifugal filter (Millipore, Billerica, MA). The filtrates were diluted 100 fold in ddH_2_O, and 25 μL of each sample was subject to HPAEC analysis. NaOH (100 mM) was used to elute the saccharides at the flow rate of 0.3 mL/min. Xylose, xylobiose, xylotriose, xylotetraose, xylopentaose, and xylohexaose served as standards.

### Sequence and structure analysis

The nucleotide and protein sequences were aligned using the BLASTn and BLASTp programs (http://www.ncbi.nlm.nih.gov/BLAST/), respectively. The nucleotide sequence was analyzed using the NCBI ORF Finder tool (http://www.ncbi.him.nih.gov/gorf/gorf/gorf.html). The sequence assembly was performed using the Vector NTI Advance 10.0 software (Invitrogen). Genes, introns, exons and transcription initiation sites were predicted using the online software FGENESH (http://linux1.softberry.com/berry.phtml). The signal peptide was predicted using SignalP (http://www.cbs.dtu.dk/services/SignalP/). The potential *N*-glycolyzation sites were predicted online (http://www.cbs.dtu.dk/services/NetNGlyc/). Multiple sequence alignments were performed with ClustalW. Homology modeling was performed by the modeler module of the Discovery Studio2.5 (DS2.5) software (Accelrys, BioVia, San Diego, CA, USA) using the GH10 xylanase from *Penicillium canescens* (PDB_id: 4F8X) [20] as a template. The Blosum-62 matrix was used with a gap penalty of 10 and a gap extension penalty of one. The modeler module of DS2.5 was used to build the three-dimensional models of Af-XYNA. The ‘number of models’ parameter was set to 10 and the remaining parameters were set to the default values. The best-fit models of the Af-XYNA were evaluated with a Ramachandran Plot using the PROCHECK program and the Verify Protein (Profiles-3D) program in DS2.5. The structure of Af-XYNA was displayed using Discovery studio visualizer software.

## Results and discussion

### Gene sequence analysis of *Af*-XYNA

**Fig 1:**
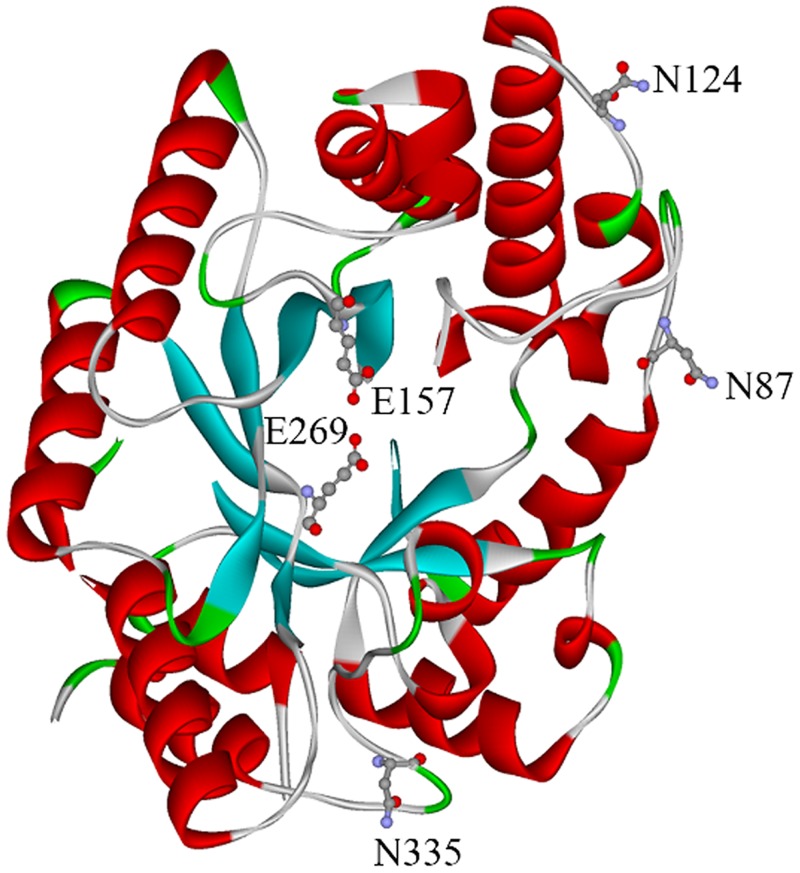
Homology modeling of Af-XYNA with the catalytic sites and N-glycosylation sites indicated.

**Fig 2:**
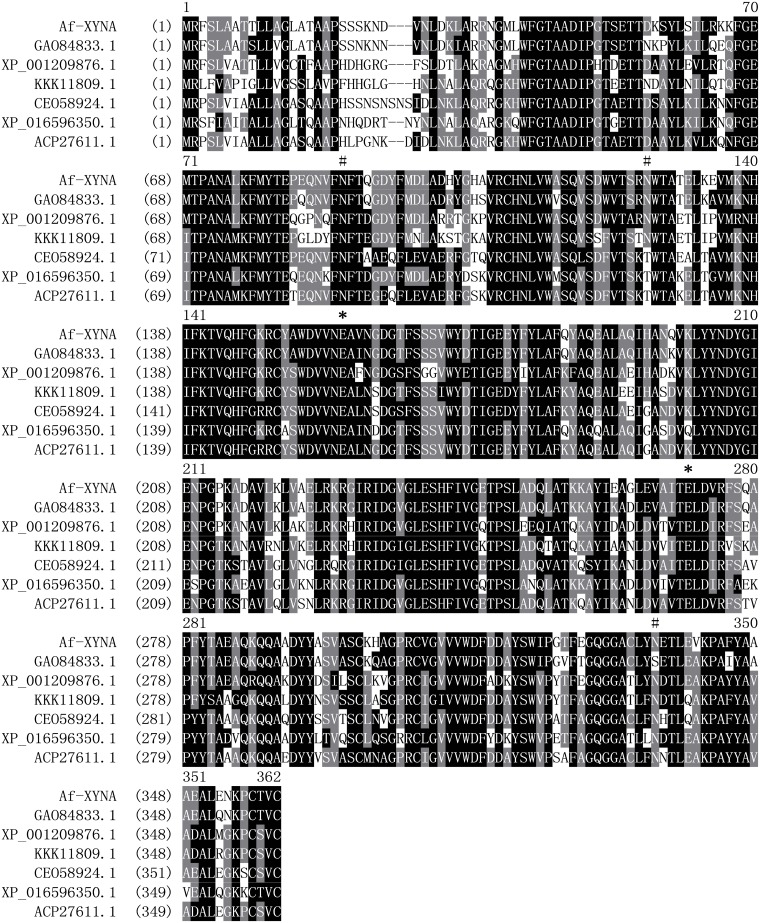
Amino acid sequence alignment of Af-XynA with the GH10 xylanases from *A*. *udagawae* (GAO84833.1), *A*. *terreus* NIH2624 (XP_001209876.1), *A*. *rambellii* (KKK11809.1), *P*. *brasilianum* (CEO58924.1), *P*. *expansum* (XP_016596350.1), and *P*. *canescens* (ACP27611.1) using the ClustalW program. Identical and similar amino acids are indicated in black and grey, respectively. The conserved catalytic residues are indicated by *. The potential N-glycosylation sites are indicated by #.

The full-length open reading frame (ORF) of *Af-xynA* (XM_749010.1) contains 1043 bp that encodes a 344 amino-acid protein with a calculated molecular weight of 38.5 kDa. The amino acid sequence of *Af*-XYNA exhibited the highest identity of 100% with a putative GH 10 xylanase from *A*. *fumigatus* Af293 (GenBank accession no. XP 754103.1) and 78% identity with a functionally characterized xylanase from *Penicillium canescens* (PDB_id 1: 4F8X). SignalP analysis revealed the existence of an *N*-terminal signal peptide at residues 1–17. Homology modeling (Fig 1) indicated the presence of two putative catalytic glutamate residues (E157 and E269) and six cysteine residues (C107, C149, C299, C306, C332, and C356) highly conserved among GH 10 members. These cysteine residues are apt to form three disulfide bonds, which are crucial for correct protein folding and maintaining the advanced structure of thermostable enzymes [21]. There are three potential N-glycosylation sites (N-87, N-124 and N-335) in Af-XYNA, and two of them correspond to Asn88 and Asn336 of 4F8X. According to the aligning result, the Asn at site 124 is conserved in xylanases from *Aspergillus*, which is replaced by Thr in *Penicillium* (Fig 2).

### Expression and purification of wild type *Af*-XYNA and its mutant form

**Fig 3:**
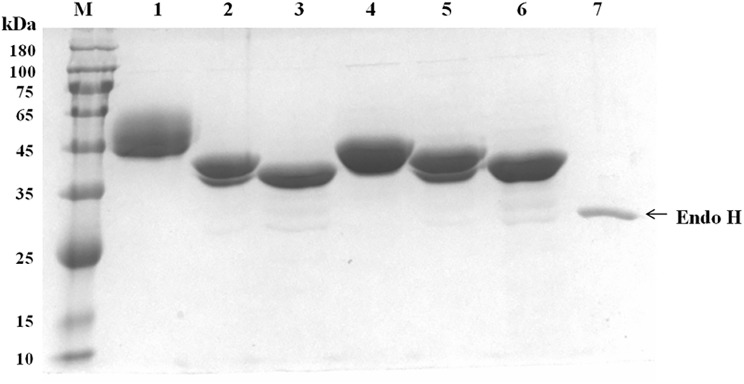
SDS-PAGE analysis of the purified recombinant Af-XynA and N124T mutant. Lanes: M, standard protein molecular weight markers; 1, the purified Af-XynA; 2, the native deglycosylated Af-XynA with Endo H; 3, the denatured deglycosylation Af-XynA with Endo H; 4, the purified N124T mutant; 5, the native deglycosylated Af-XynA with Endo H; 6, the denatured deglycosylation Af-XynA with Endo H; 7, EndoH.

The gene fragments of *Af-xynA* and *N124T* without the signal peptide-coding sequences were expressed in *P*. *pastoris*. Positive transformants were screened for enzyme activity after induction with methanol for 72 h by using the DNS method. The results indicated that the genes were successfully expressed in *P*. *pastoris* and encoded functional xylanases. The recombinant xylanases were easily purified to electrophoretic homogeneity by anion-exchange chromatography. SDS-PAGE analysis indicated that purified Af-XYNA yielded a main band of 50.0 kDa with faint smear, while N124T had an apparent molecular mass of about 43.0 kDa (Fig 3). Both enzymes showed a molecular mass higher than the calculated ones (38.5 kDa). After digestion with Endo H, the enzyme molecular masses decreased to approximately 39.0 kDa. The results showed that glycans at N124 accounts for the majority of glycosylation.

It has been reported that N-glycosylation at different positions has different effects on protein secretion and catalytic activity [17]. For instance, the glycosylation at N224 of β-glucosidase from *A*. *terreus* and at N97 of GH45 β-1,4-endoglucanase from *Apriona germari* are unnecessary for secretion but essential for enzyme activity [22, 23]. The enzymatic activity is generally decreased upon removal of one or more N-glycans [17] with some exceptions. For example, the human protein C without the glycosylation site at N97 showed a 2–3-fold increase in anticoagulant activity; and the activity of deglycosylated β-glucuronidase from *P*. *purpurogenum* increased by 20–70% compared with the glycosylated one [24, 25]. Furthermore, the effect of glycosylation at different sites on the substrate digestion rates, secretion and enzyme activity has aroused extensive interests. The N-60 glycan can influence the secretion and proper translation of *Rhizopus chinensis* lipase, while N-48 had no effect [26]. After deleting the glycan on N-45 of cellobiohydrolase I (Cel7A) from *P*. *verruculosum*, the mutant showed an increase in the hydrolysis rate of Avicel and milled aspen wood, but mutants N194A and N388A had no changes [27]. Therefore, the glycosylation at various degrees and different sites may play different roles in the local conformation, mobility and stability of a protein.

### Enzymatic properties of Af-XYNA

**Fig 4:**
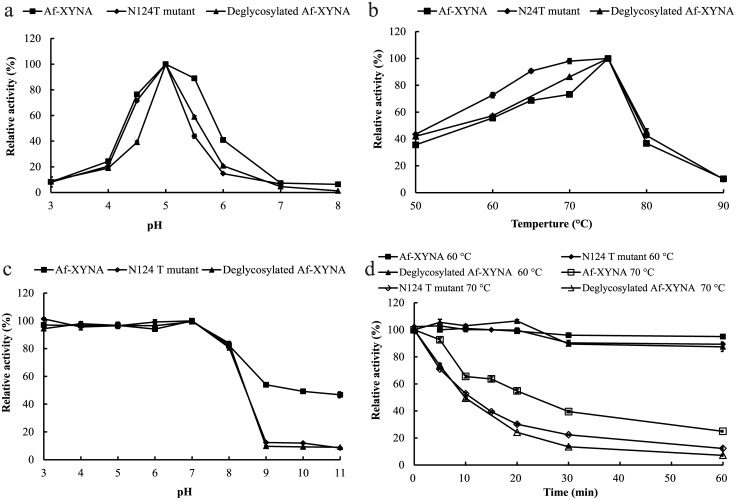
Characterization of the purified recombinant Af-XynA, mutant N124T, and deglycosylated Af-XynA (50 μg/ml). **A** pH curve; **B** temperature curve; **C** pH stability; **D** thermal stability.

The optimal pH of wild-type Af-XYNA was pH 5.0, and more than 44% activity was retained over the pH range of 4.0 to 7.0 (Fig 4a). This property is similar to other reported GH 10 xylanases from fungi [4], such as EX1 from *Trichoderma* sp. K9301 [28], XYN10G5 from *Phialophora* sp. G5 [29], and a xylanase 10 from *A*. *terreus* BCC129 [30]. However, some xylanases from *Humicola insolens* [31], *A*. *nidulans* KK-99 [32], and *Chrysosporium lucknowense* [33] showed alkali-tolerant property. Af-XYNA remained >69% of the relative activities at pH 3.0–8.0, and retaining more than 46% of the initial enzyme activities even at pH 9.0–11.0 (Fig 4c). So far most known GH 10 fungal xylanases have been reported to be more active and stable at acidic to neutral pH range (2.0–8.0) [34].

Af-XYNA showed optimal activity at high temperature (75°C; Fig 4b), which is much higher than most xylanases from mesophilic fungi, which had the optimal temperatures between 40 and 70°C [35]. A few xylanases from thermophilic fungus *Thermoascus aurantiacus* CBMAI 756 (75°C), Xyn10B and Xyn10C from *Chrysosporium lucknowense* (80–85°C), and XYL10C from *Bispora* sp. MEY-1 (85°C) have higher temperature optima than Af-XYNA [36–37]. In addition, Af-XYNA was highly stable at 60°C, retaining >80% activity after 1 h incubation at 60°C without substrate, but lost activity rapidly when treated at 70°C (Fig 4d). Some fungal xylanases have high temperature optima (≥ 70°C), such as XynA from *Phanerochaete chrysosporium* [38], XYN10G5 from *Phialophora* sp. G5 [29], and Xyl Ia and Ib from *Myceliophthora* sp. IMI 387099 [39]. However, these xylanases are less stable at 70°C. The reason might be that the presence of substrate in the activity assay could partially stabilize the enzyme conformation, while no substrate was added during the incubation processes of thermostability assay [40].

The activities of Af-XYNA were not inhibited by K^+^, Cr^3+^, Mg^2+^, Fe^3+^, Na^+^, Co^2+^ and Ca^2+^ substantially (Table 1). Partial inhibition of the enzyme activity was detected in the presence of Cu^2+^, Ag^+^ and EDTA. But β-mercaptoethanol enhanced the activity observably. Like most fungal xylanases sensitive to SDS and Zn^2+^, analysis of the effect of two regents at 5 mM concentration on xylanse activity showed that the residual enzyme activities were 18.4 and 18.7, respectively. The *K*_m_, *V*_maxt_ values of purified Af-XYNA using birchwood xylan as the substrate was 1.3 ± 0.1 mg/mL and 268 ± 7 μmol/min/mg, respectively.

**Table 1:** Effects of metal ions and chemicals on the activity of purified recombinant Af-XYNA.

Chemicals	Relative activity (%)^a^	Chemicals	Relative activity (%)
None	100.0 ± 1.6	CrCl_3_	100.2 ± 1.4
Pb(CH_3_COO)_2_	92.9 ± 1.6	CoCl_2_	101.8 ± 2.2
MnSO_4_	97.9 ± 3.7	ZnSO_4_	18.7 ± 4.1
CaCl_2_	99.3 ± 1.3	CuSO_4_	94.5 ± 1.4
FeCl_3_	100.4 ± 3.1	HgCl_2_	0
MgSO_4_	103.5 ± 3.8	AgNO_3_	79.9 ± 1.0
KCl	103.9 ± 1.6	EDTA	86.9 ± 2.5
NaCl	104.0 ± 2.3	β-Mercaptoethanol	181.1 ± 1.2
NiSO_4_	104.1 ± 1.1	SDS	18.4 ± 3.9

^a^ Values represent the means ± SD (n = 3) relative to the untreated control samples

### Substrate specificity and analysis of hydrolysis products

Purified recombinant Af-XYNA was most active on birchwood xylan (100%), moderate on beechwood xylan (95.1%) and soluble wheat arabinoxylan (125.8%). And, no activity was detected in the presence of barley β-glucan, CMC-Na, and locust bean gum. Thus, the cellulose activity-free Af-XYNA may represent a candidate for biobleaching of paper pulp.

The hydrolysis products of birchwood xylan and soluble wheat arabinoxylan by Af-XYNA were analyzed by HPAEC. Af-XYNA had ability to hydrolyze two xylan substrates into a mixture of xylose and xylobiose with xylobiose as the major hydrolysis product (88%). Xylooligosaccharides, especially xylobiose, have ability to stimulate the growth of intestinal bifidobacteria and promote a favorable intestinal environment [41]. Thus, application of Af-XYNA to produce xylobiose from soluble wheat arabinoxylan is economic and attractive.

### The contribution of N-glycan to the enzymatic characteristics of Af-XYNA

To investigate the role of the N-glycans attached to Af-XYNA, we compared the enzymatic activity of WT with its deglycosylated counterpart (DE) treated and mutant N124T. The deglycosylated DE and N124T showed similar pH (5.0) and temperature (75°C) optima to WT (Fig 4a and 4b). However, DE and N124T exhibited pH adaptation to a narrower range. WT showed > 76% of its maximal activity between pH 4.5 and 5.5 while DE and N124T were both below 50%. Besides, DE and N124T had worse pH stability, and only retained 5% relative activities after treatment at pH 9.0–11.0 for 1 h (Fig 4c). These pH properties were similar with the GH 10 xylanase 4F8X from *P*. *canescens*, which had no N-glycosylation site at T124. These results showed the N-glycan at N124 plays a key role in pH adaptation and stability of Af-XYNA.

The enzymes had good stability at 60°C. When the enzymes were incubated at 70°C for 1 h, WT remained about 25% activity, while N124T and DE only retained 12% and 7% activities, respectively (Fig 4d). It indicated that the thermostability of DE and N124T was worse at high temperature than that of WT. The thermodynamic analysis indicated that WT had a *T*_m_ of 63.9°C, which was 1.8°C higher than N124T (62.1°C). This decrease in stability confirmed the above-described results and suggested that N-glycan at N124 made remarkable contribution to the improvement of thermostability. This observation is in accordance with the theory that glycosylation could maintain the stability of protein [42]. Steric interactions between the sugar residues and protein structure may enable the stabilization of a protein. Steric effects of glycosylation rather than the length of N-glycan may account for the increase in thermostability, and more dispersed glycosylation patterns favors stabilizing protein-glycan interactions [43]. According to the characters of economic benefits, the thermostability can be used widely in industrial applications of specific requirements.

Compared with WT (251 ± 10 U/mg), the specific activity of DE and N124T were basically decreased towards birchwood xylan (204 ± 6 U/mg and 220 ± 5 U/mg). In addition, the *K*_m_, and *V*_max_ values of purified N124T were 1.9 ± 0.1 mg/ml, 241 ± 8 μmol /min/mg, respectively, which were similar to that of WT. The results indicated that the glycans at N124 did not reduce the substrate affinity of Af-XynA, which corresponds to the fact that N124 is far from the catalytic domain.

## Conclusions

A xylanase gene (*Af-xynA*) was cloned from *A*. *fumigatus* and expressed in *P*. *pastoris*. Recombinant Af-XynA associated with N-glycosylation showed maximum activity at pH 5.0 and 75°C, and had good thermostability at 60°C. These favorable enzymatic properties make Af-XynA a good candidate for application in the animal feed industry. By removing the N-glycosylation with endo H or mutating the N-glycosylation site N124, the modified enzymes showed narrower pH adaptation ranges, worse pH/thermal stability and lower activity. This study demonstrated that the N-glycosylation at different degrees and sites had diverse effect on the enzyme properties, and provided theoretical support for the improvement of enzyme stability.

## Abbreviations

DE: deglycosylated enzyme
DNS: dinitrosalicylic acid assay
DSC: differential scanning calorimetry
Endo H: endo-β-*N*-acetylglucosaminidase H
GH: glycoside hydrolase
HPAEC: high-performance anion exchange chromatography
LB: Luria-Bertani medium
SDS-PAGE: sodium dodecyl sulfate polyacrylamide gel electrophoresis
WT: wild-type
YPD: yeast peptone dextrose medium
